# Total proctocolectomy with end ileostomy for acute onset of ulcerative colitis during chemoradiotherapy for lung adenocarcinoma (successfully treated by surgery): a case report

**DOI:** 10.1186/s40792-020-00886-x

**Published:** 2020-06-01

**Authors:** Koichi Mohri, Kazuhiro Hiramatsu, Yoshihisa Shibata, Taro Aoba, Masahiro Fujii, Atsuki Arimoto, Akira Ito, Takehito Kato

**Affiliations:** Department of General Surgery, Toyohashi Municipal Hospital, 50 Hachiken-nishi, Aotake-cho, Toyohashi, Aichi 441-8570 Japan

**Keywords:** Ulcerative colitis, Lung adenocarcinoma, Chemoradiotherapy, Total proctocolectomy

## Abstract

**Background:**

Ulcerative colitis (UC) developing during chemotherapy is very rare. Here, we describe a case of acute onset during chemoradiotherapy for lung adenocarcinoma, requiring a total proctocolectomy.

**Case presentation:**

A 52-year-old man was admitted to the hospital for chemoradiotherapy of lung cancer. He had no obvious history of gastrointestinal diseases, and concurrent chemoradiotherapy was initiated. Thirteen days after 2 cycles of cisplatin and vinorelbine, he experienced persistent hematochezia. Findings of the colonoscopy revealed edematous thickening from the rectum to the transverse colon, suggesting UC, drug-induced colitis, or infectious colitis. Results from bacterial culture were negative for *Clostridium difficile* and methicillin-resistant *Staphylococcus aureus* (MRSA). Immunohistological staining for cytomegalovirus was also negative. Although he was clinically diagnosed with UC and treated with intravenous glucocorticoid, his symptoms gradually worsened and an abdominal X-ray revealed megacolon. Thirty-five days after conservative therapy, a total proctocolectomy with end permanent ileostomy was performed. Based on pathological findings and clinical course, he was diagnosed with UC.

**Conclusion:**

Although the pathogenesis of UC during chemotherapy has been unknown, chemotherapy could be one of the causes of UC in this case. UC should be included in the differential diagnosis in patients with progressive colitis during chemotherapy.

## Background

It has been reported that cytotoxic chemotherapy can cause various adverse effects on the gastrointestinal tract [1]. Although medication-induced colitis is a common phenomenon, chemotherapy-associated ulcerative colitis (UC) is very rare and only a few cases have been reported. Here, we describe a case of acute onset UC during chemoradiotherapy in a patient with non-small cell lung adenocarcinoma, requiring a total proctocolectomy with end ileostomy.

## Case presentation

**Fig. 1:**
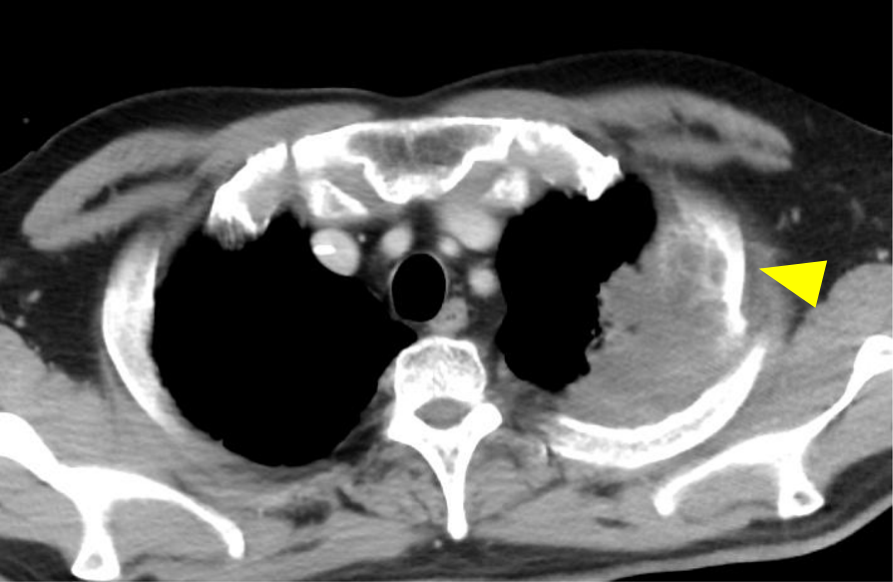
Thoracic CT scan. CT revealing an 80 × 60 mm mass lesion at the left upper lobe, which invaded the second rib (arrowhead)

A 52-year-old man was admitted to a previous hospital for chemoradiotherapy for lung adenocarcinoma. He had no obvious history of gastrointestinal diseases and no family history of inflammatory bowel diseases. A computed tomography (CT) scan revealed an 80 × 60 mm mass lesion at the left upper lobe, invading the second rib (Fig. 1). Specimens obtained by transbronchial biopsy diagnosed a non-squamous non-small cell lung cancer. Immunohistochemical staining results revealed no gene mutations (EGFR, KRAS, ALK, BRAF, ROS1, and HER2) and low PD-L1 expression.

**Fig. 2:**
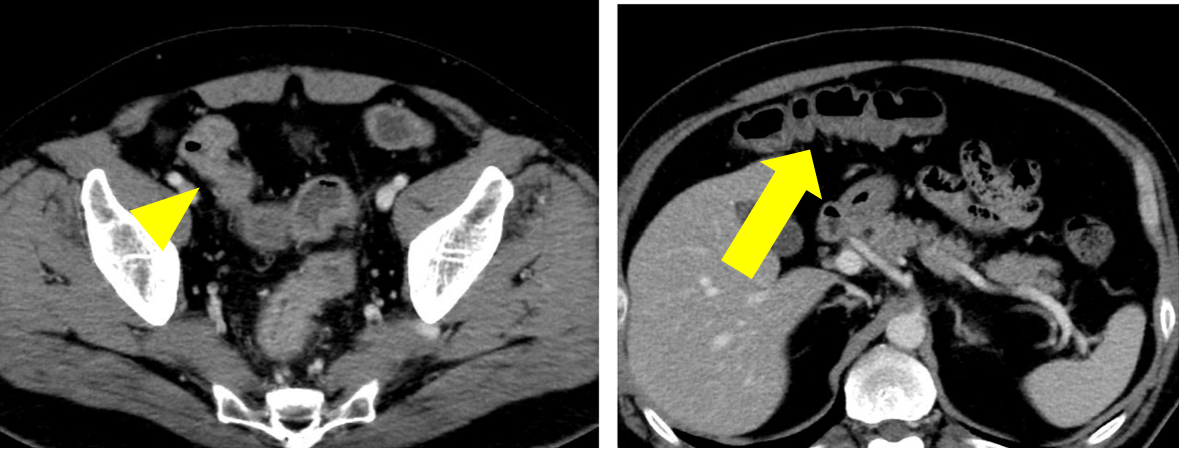
First abdominal CT scans. CT revealing edematous thickening bowel from the rectum to the transverse colon (arrow). The appearance of the cecum is normal (arrowhead)

**Fig. 3:**
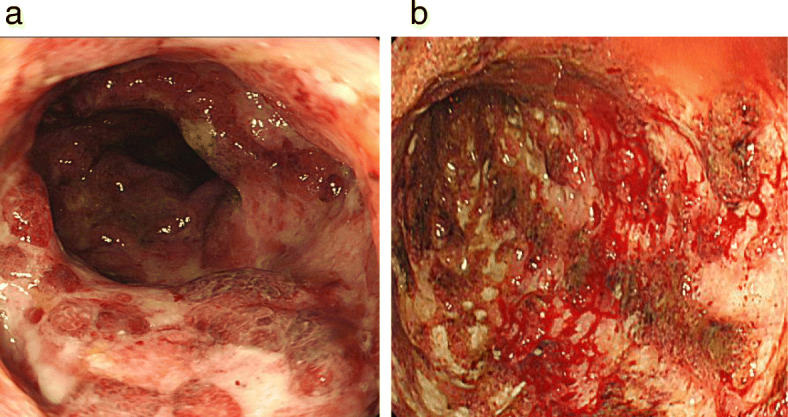
Colonoscopy images. **a** Colonoscopy revealing extensive ulceration of the mucosa. The surface is irregular and erythematous with loss of the normal vascular markings. **b** Extensive pseudopolyps in the rectum

**Fig. 4:**
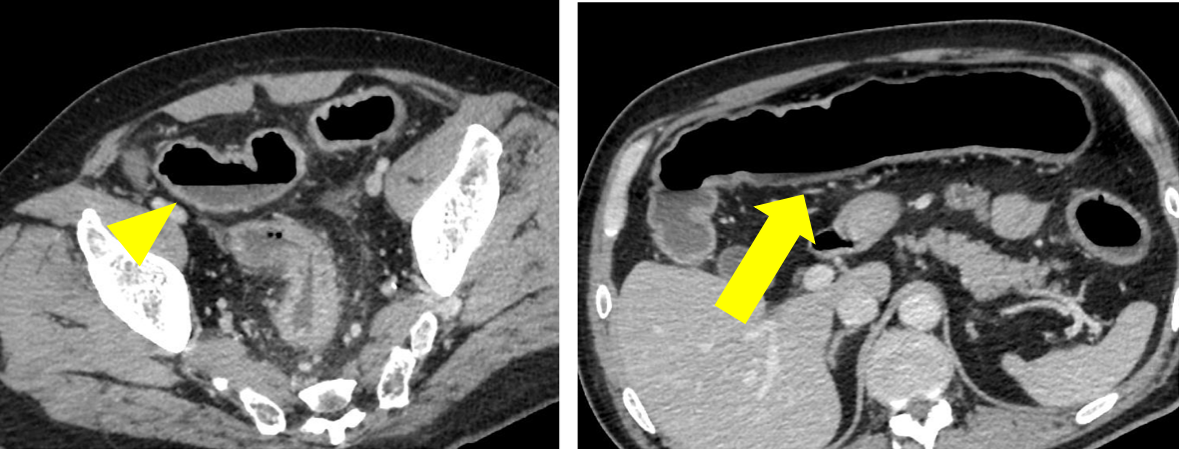
Second abdominal CT scans. CT revealing extensive edema and dilated lumen without haustra in the entire colon; transverse colon (arrow) and cecum (arrowhead)

**Fig. 5:**
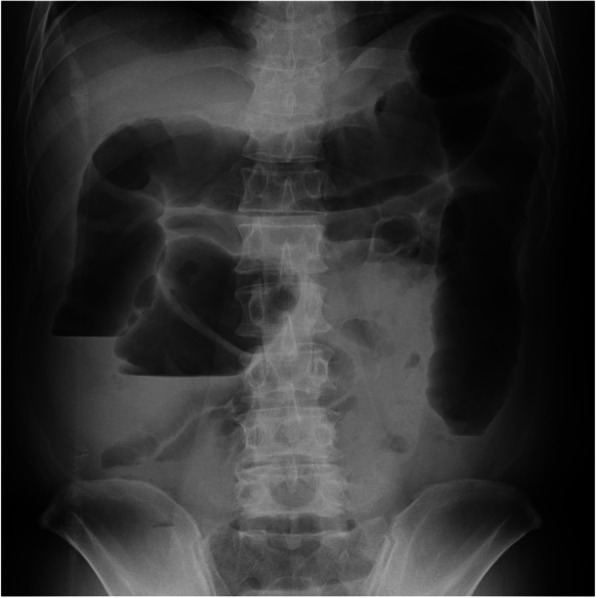
Abdominal X-ray. Megacolon is evident

**Fig. 6:**
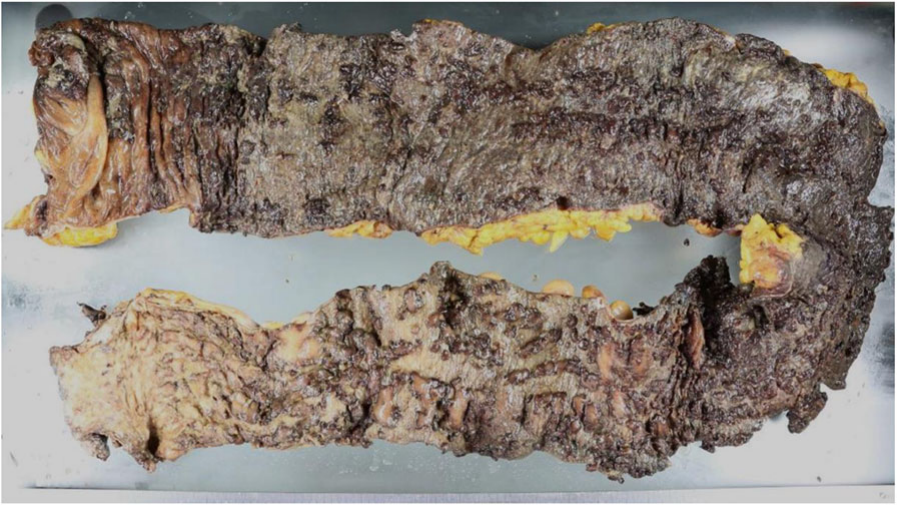
The resected specimen shows pseudopolyps with multiple deep ulcerations from the dentate line to the cecum

**Fig. 7:**
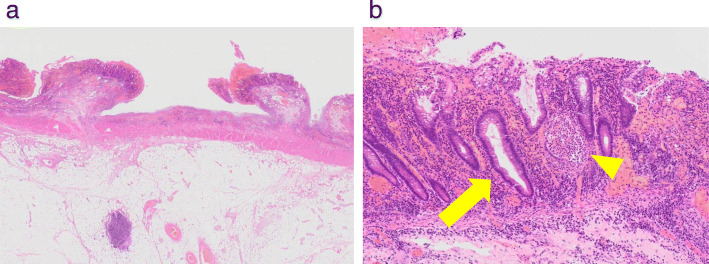
**a** Macroscopic appearance of the resected specimens showing pseudopolyps. HE staining (× 6). **b** Microscopic findings showing goblet cell depletion (arrow), crypt abscesses (arrowhead), and infiltration of polymorphonuclear cells. HE staining (× 60)

The clinical stage was T4N0M0 and stage IIIA, and concurrent chemoradiotherapy was initiated. Thirteen days after 2 cycles of cisplatin and vinorelbine, he experienced grade 2 abdominal pain, diarrhea, and hematochezia based on the National Cancer Institute Common Toxicity Criteria (version 3.0 of the toxicity scale). Although radiotherapy was completed (60 Gy/30 Fr), concurrent chemotherapy was discontinued. Intravenous antibiotics demonstrated poor efficacy in treating his symptoms. A CT revealed an edematous thickening of the bowel from the rectum to the transverse colon (Fig. 2). He underwent a colonoscopy, which revealed an edematous bowel wall with ulceration and erythema extending from the rectum proximally to the transverse colon in a continuous and circumferential pattern (Fig. 3a). Findings of the colonoscopy were suggestive of UC, drug-induced colitis, or infectious colitis. Results from bacterial cultures of his stool and mucosa specimens were negative for the presence of the *Clostridium difficile* (CD) and methicillin-resistant *Staphylococcus aureus* (MRSA). Immunohistological staining for cytomegalovirus (CMV) from a colon tissue biopsy was also negative. Pathological findings from rectal biopsy revealed infiltration of inflammatory cells including plasmacytes, but no crypt abscesses; therefore, it was still difficult to distinguish between drug-induced colitis and UC. Following 28 days of conservative therapy, he was transferred to our hospital, and blood tests showed elevated inflammatory biomarkers (white blood cell count, 12,880/μL; hemoglobin, 10.0 g/dL; C-reactive protein, 23.1 mg/dL; serum albumin, 2.3 g/dL). The CT scan showed pancolitis with extensive edema and a dilated lumen without haustra in the entire colon (Fig. 4). Sigmoid colonoscopy showed extensive multiple ulceration revealing pseudopolyps in a continuous pattern (Fig. 3b). He was clinically diagnosed with pancolitis-type UC and treated with intravenous glucocorticoids (prednisolone, 90 mg). His symptoms gradually worsened, and an abdominal X-ray revealed megacolon (Fig. 5). After 7 days of administration of prednisolone, total proctocolectomy with permanent end ileostomy was performed. Laparotomy revealed a significantly dilated colon. There were penetrations on some parts of the abdominal wall. The surgical duration was 229 min, and the total blood loss was 251 mL. His postoperative course was uneventful, and he was discharged on the 14th postoperative day. The resected specimen revealed the appearance of pseudopolyps with multiple deep ulcerations (Fig. 6). Histological examination demonstrated goblet cell depletion, crypt abscesses, and infiltration of polymorphonuclear cells (Fig. 7). There were no other malignant findings or atypical lesions. UC was diagnosed based on the pathological findings and clinical manifestations and course of the disease. Ten months after the surgery, there were no signs of recurrence. Maintenance therapy with durvalumab was not initiated, considering the risk of enteritis and extraintestinal manifestations of UC.

## Discussion

UC is a diffuse specific inflammatory condition limited to the mucosal layer of the colon. It is often characterized by relapsing and remitting episodes. Bowel inflammation always extends from the rectum to more proximal portions of the colon in a continuous fashion [2]. Patients with UC repeatedly present with bloody diarrhea. The onset of symptoms is usually gradual and progresses over several weeks. The severity of symptoms may range from mild to severe disease with frequent stools and continuous bleeding [3]. The endoscopic findings of UC include a loss of vascular markings, erosions, macro-ulcerations, and pseudopolyps characteristically beginning at the anal verge and extending proximally. The inflammatory lesion is almost always continuous and circumferential. Pseudopolyps are not specific for UC, but are more common in severe UC, occurring in approximately 20% of cases [4]. The pathological findings suggestive of UC include crypt abscesses, epithelial cell abnormalities, and lamina propria cellularity. Although none of these features are specific for UC, the presence of two or more histological findings is highly suggestive [5, 6].

Patients with mild to moderate UC receive medical treatments, while those with UC who develop life-threatening complications including colonic perforation, massive gastrointestinal hemorrhage, and toxic megacolon may require emergency surgery [7]. Toxic megacolon is characterized by a colonic diameter of ≥ 6 cm or a cecal diameter of > 9 cm and the presence of systemic toxicity [8]. This case was a fulminant form of UC that developed during chemoradiotherapy for lung cancer. A proctocolectomy with end ileostomy was performed in view of the likelihood of fewer complications and a complete cure for severe colitis.

Medication-induced colitis is one of the differential diagnoses of UC, which is common with the use of cytotoxic anticancer agents including fluoropyrimidines, irinotecan, methotrexate, docetaxel, vinorelbine, and cisplatin [1]. Medication-induced colitis is categorized into four groups: ischemia, colonic pseudo-obstruction, neutropenic colitis (infection), and drug allergy [9]. In particular, chemotherapy is associated with neutropenic colitis. Its pathogenesis probably involves a combination of factors, including mucosal injury by cytotoxic drugs, altered microbial flora, and weakened immunologic defenses to virulent micro-organism [10]. Medication-induced colitis is generally recognized by prompt clinical improvement on cessation of medication. In our case, we confirmed negative results for the presence of *C. difficile*, MRSA, and CMV.

UC developing during chemotherapy is rare; however, some cases have been reported. Fujita et al. reported a patient with lung cancer whose UC was induced by cisplatin and vinorelbine therapy; the authors speculated that intestinal mucosal vasculopathy associated with vinorelbine may affect the development of UC [11]. Tanaka et al. reported a patient with lung cancer whose UC was induced by bevacizumab and required subtotal colectomy [12]. Tomioka et al. reported a case of UC after chemotherapy and colorectal resection. The authors considered that four factors including chemotherapy, colorectal operative stress, changes in blood circulation because of surgery, and mental stress are responsible for the onset of UC [13].

Evidence from several studies suggest that UC was induced by chemotherapy in this case. First, the patient had no history of gastrointestinal diseases including inflammatory bowel diseases. It is also unlikely that his pre-existing UC developed rapidly during chemoradiotherapy. Second, fulminant colitis developed during chemoradiotherapy, and the clinical symptoms worsened after chemotherapy was discontinued. Third, other differential diseases were clinically and pathologically found to be negative. Ischemic colitis is often located on the left side of the colon, and a segmental distribution and abrupt transition between injured and non-injured mucosa on colonoscopy are suggestive [14].

The pathogenesis of UC during chemotherapy has not been clarified. Although the pathogenesis underlying the causes of UC remains unknown, some mechanisms were speculated. Infections and the immune response have been implicated in the pathogenesis of UC. Many studies suggest dysregulated immune responses to microbes within the intestinal lumen [15]. Damage to the intestinal mucosa caused by anticancer drugs is probably caused by dysregulation of the immune system and is involved in the development of UC.

There are some limitations to our report. The mechanism between the administration of chemotherapy and the onset of UC was not completely elucidated; the etiology of UC needs to be defined more precisely. Second, the pathological findings of UC may be similar to those of drug-induced enteritis; therefore, the clinical course and colonoscopic findings are also important. This case may demonstrate some overlap between chemotherapy-induced colitis and UC.

## Conclusions

Although the pathogenesis of UC during chemoradiotherapy has remained unknown, chemotherapy was probably one of the causes of the acute onset of UC in this case. Careful clinical and histopathological examinations are important for identifying such cases. UC should be included in the differential diagnosis when encountering patients with progressive colitis during chemotherapy.

## Data Availability

Data sharing is applicable to this article.

## Abbreviations

CD: *Clostridium difficile*
CMV: Cytomegalovirus
CT: Computed tomography
MRSA: Methicillin-resistant *Staphylococcus aureus*
UC: Ulcerative colitis
